# A qualitative exploration of the potential benefits of Nature-based Approaches for staff and Children and Young People in Child and Adolescent Mental Health Services

**DOI:** 10.1371/journal.pmen.0000389

**Published:** 2025-07-28

**Authors:** Beth Chapman, Siobhan B. Mitchell, Rebecca Hardwick, Hélène Bonnici, Hazel Banks, Silvana Mareva, Rachel Hayes

**Affiliations:** 1 Cornwall Partnership NHS Foundation Trust, Cornwall, United Kingdom; 2 NIHR Applied Research Collaboration South West Peninsula (PenARC), University of Exeter, Exeter, United Kingdom; 3 NIHR Applied Research Collaboration South West Peninsula (PenARC), University of Plymouth, Plymouth, United Kingdom; 4 Faculty of Health and Life Sciences, University of Exeter, Exeter, United Kingdom; Universiti Brunei Darussalam Pengiran Anak Puteri Rashidah Sa’adatul Bolkiah Institute of Health Sciences, BRUNEI DARUSSALAM

## Abstract

Across the UK there are concerns about young people’s mental health, with rates of ill health increasing and referrals to Child and Adolescent Mental Health services (CAMHS) doubling. This study explores the potential benefits of incorporating Nature-Based Approaches (NBAs) within CAMHS. Providers are understaffed, under-resourced and under pressure. NBAs offer a way of working which could address some of these challenges, yet little is known about the potential benefits of NBAs in the context of CAMHS. The project aimed to explore staff understanding of NBAs, and to identify potential benefits of integrating NBAs into practice. Staff within a South of England CAMHS service were asked to complete a survey and qualitative interview to explore staff perspectives on using NBAs in their practice. Staff were also given the opportunity to attend a nature-based training course. All participants were sampled from one NHS Trust and the study was open to all staff whether or not they had attended the training. Data were synthesised to produce an understanding of staff attitudes towards NBAs and the potential benefits of this approach. Ninety-seven staff responded to the survey, and fourteen staff members were interviewed. Data synthesis generated three themes: Tension between the culture of CAMHS and NBAs (Theme 1) and the need for buy-in and governance support (Theme 2), whilst Theme 3 describes the potential benefits of NBAs for staff and service users in CAMHS and is the focus of this paper. This study highlights the perceived benefits of adopting NBAs for both CAMHS service users and staff. Participants noted advantages including increased choice, individualisation, enhanced therapeutic quality, and new ways of working that offer greater autonomy, creativity, and flexibility at individual and service level. Further research is recommended to explore the full potential of NBAs to enhance service user and staff experiences in CAMHS.

## Background

Across the UK there are concerns about young people’s mental health, with rates as high as 1 in 5 children and young people (CYP) in England having a probable mental health disorder [1–3]. Referrals to Child and Adolescent Mental Health services (CAMHS) have doubled since before the Covid-19 pandemic [3], and there are three times as many CYP in contact with mental health services now than there were seven years ago [4]. Providers are understaffed, under-resourced and under pressure [5].

Thresholds for getting help through specialist CAMHS have increased to try to manage demand [6]; CYP in CAMHS are therefore presenting with higher levels of complexity, risk and need. There is a lack of data around how complexity or severity of symptoms may act as prognostic predictors. However, those with higher levels of mental health difficulty have been shown to have lower levels of improvement at follow up [7]. Moreover, those from areas with higher levels of deprivation are more likely to be in contact with mental health services [5].

Nature-based approaches (NBAs) are one way in which social determinants affecting people’s health are being addressed. This umbrella term refers to any aspect of health support and services which are provided within a natural environment, such as urban nature (parks, gardens, hospital grounds), agricultural nature, woodlands and wild nature, including blue space such as sea, lakes and rivers. Evidence suggests that NBAs may promote wellbeing through increasing nature connectedness [8,21,23,26], but little is known about the potential benefits of this approach in the context of CAMHS services.

Underpinning our understanding of the potential benefits of NBAs are several theories, which have been developed to explain the connections between nature and human health and wellbeing. The Biophilia hypothesis proposes that humans have an innate tendency to seek a relationship with nature [9,10]. The case for a biological affinity with nature is interwoven with other theories, such as Attention Restoration Theory and Stress Reduction Theory [11,12]. The former describes the restorative benefit for attention associated with the undemanding soft focus or fascination that occurs when in a natural environment, such as watching leaves blowing in the wind or birds feeding [13]; and the latter describes the notion that the natural environment can promote physiological recovery from stress while an urban or less natural environment may trigger stress [12]. Nature-based approaches can contribute to recovery from acute stress and buffering against chronic stress [14]. Objective physiological measures such as heart rate, blood pressure and cortisol levels, and subjective measures, including perception of stress, support this association between exposure to nature and stress reduction [15].

Traditionally, NBAs have not extended into specialist mental health services but have remained within the domain of social prescribing. A recent report from the government recognises that these third sector providers often cannot offer meaningful or appropriate support to people with more complex mental health needs due to a lack of training and resources [16]. Making use of nature-based approaches within specialist mental health services could help to address this inequity and aligns with the Royal College of Psychiatrists 2023 Net Zero Mental Health Care Guidance and the Greener NHS principles of low carbon care [17].

Nature-based approaches meet the definition of complex interventions, due to the flexibility in the mode and content of delivery, the context in which they are delivered, and the intended recipient [18]. Outcomes of NBA’s traverse multiple domains including health, wellbeing, social, emotional and behavioural and can be experienced differently by individuals. It follows that there are also multiple underlying and interacting mechanisms through which nature can exert its effect [19]. Specifically, nature connectedness has been recognised as a construct which relates strongly to wellbeing. Nature connectedness quantifies a person’s sense of their relationship with nature across five pathways: Senses or contact, beauty, emotion, meaning and compassion [20]. While both time in nature and connectedness to nature are positively predictive of better general physical health, the strength of nature connectedness and time spent engaging with nature through simple activities has a higher predictive value of wellbeing (hedonic wellbeing, e.g., happiness and eudaimonic wellbeing, e.g., self-realization, fulfilment) and illbeing (anxiety and depression) [21].

Compared to earlier childhood, adolescents report a reduction in how connected they feel to nature, with rates dropping by 30% from age 9–15 [22]. Benefits of nature connectedness specifically for children and young people’s mental health, such as emotional wellbeing, stress and health-related quality of life, have been identified in systematic reviews [23,24]. Moreover, these reviews identify particular patient groups who may benefit, for example, CYP with behavioural difficulties including hyperactivity and inattention problems [23,24]. Surveys suggest a direct link between nature connection and pro-environmental and pro-conservation behaviours, which could be a crucial step to redressing the threat to health due to the loss of nature, especially in a generation that globally experiences high levels of climate anxiety [25,26]. Less is known about the potential benefits of nature-based approaches in a broader sense within mental health services.

Nature could be utilised as a specific therapeutic tool or approach in itself, or integrated throughout clinical practice, for example, bringing nature into existing clinical spaces, such as having plants, natural sounds, images and objects in the waiting and therapy rooms. This would promote incidental contact with nature, as would holding treatment as usual in an outdoor space, which increases time spent in nature. While evidence around the use of targeted NBAs within CAMHS is currently limited, offering services outside of the traditional clinic-based environment may not only be beneficial to children and young people’s wellbeing, but it may further improve our understanding of more age-appropriate and personalised care options. Hunt and colleagues explored the use of NBAs in a CAMHS inpatient unit setting: staff identified several benefits for service users, including a perception of better emotional regulation, enhancement of the therapeutic relationship, and a more sustainable approach which could foster a more active role in the recovery process beyond discharge [27]. Approaches that increase nature connectedness through the environmental context of delivery in this age group could be a valuable addition to ill health prevention and management strategies [8].

In addition to the potential positive impact on children and young people’s mental health, early evidence suggests that spending time in nature may also contribute to preventing staff burnout. Psychiatric illness, including stress, anxiety, burnout and depression, continues to be the most reported reason for sickness absence in the NHS [3,27–29]. Concurrently, issues with staff retention have been reported, with record numbers of staff reporting having left their NHS job in 2022 [30]. Moreover, NHS staff are 50% more likely to report high levels of work-related stress than the general working population [31]. This affects not only individuals but also organisational level care and financial performance; workforce planning can therefore focus on both recruitment and retention of staff [20].

In the workplace, nature contact can range from a window with a view and plants in the office to taking a lunchtime walk, or a purposeful nature connection activity such as noticing birdsong or touching nature. In the general workforce, research supports the positive effects of nature contact on mental health and cognitive ability, and reduction in perceived stress and generalised health complaints [32–34]. A systematic review of the impact of nature exposure on people who usually work indoors, concluded that indoor nature exposure can contribute to social sustainability through its impact on workers’ health and motivation. While outdoor nature exposure contributes to economic, environmental and social sustainability through its impact on workers’ restoration, stress reduction and stress coping [35].

Relatively little is known about supporting staff wellbeing through nature-based approaches in the healthcare context. Mental Health clinicians interviewed in Australia identified that being in nature could be therapeutic, relaxing and refreshing and many encouraged it, but their focus was on patient benefit [36]. The reflections of eight CAMHS inpatient staff from Oxford Health who were trained as NatureWell Facilitators included that the training itself offered enhanced peer support simply by being in a different environment [27]. Going outside of the work building helped with feelings of burnout and encouraged a fresher approach on return [27]. The practical challenges of implementing NBAs in a CAMHS setting has received some attention, elucidating organisational and cultural barriers such as the tension between the culture of CAMHS and NBAs, including the promotion of positive risk-taking associated with NBAs, perceived lack of evidence, and uncertainty around the form and function of NBAs in this setting [37]. This research also highlighted potential facilitators centred around buy-in and governance support, which may help to mitigate these barriers, such as harnessing the role of firsthand experience, involvement of a range of stakeholders in the adoption and implementation process, and demonstration of support from management.

Current evidence suggests that delivering nature-based approaches may be a way to support the health and wellbeing of both patients and staff. However, beyond the evidenced health benefits documented in the wider population, we have relatively little information about the different ways in which CAMHS staff perceive the potential benefits from these approaches. This paper seeks to explore staff views from across one CAMHS service on how working in a nature-based way could benefit both staff and patients.

## Method

Interview and free text survey data are utilised in this multimethod qualitative study looking at Nature-Based Approaches in CAMHS to address the research question: Do CAMHS staff consider that nature-based approaches may be beneficial to service users and in what ways and circumstances?

### Ethics statement

The research project was granted ethical approval from HRA (23/HRA/0191). The research was conducted in accordance with the Declaration of Helsinki. Informed consent to participate was given by all participants.

### Design

#### Training.

Training courses took place independently of the research study. Two Introductory Training away days were offered to all CAMHS staff in the Trust, with 64 attending in total. This was co-facilitated by CAMHS clinician leads for this project (CAMHS Goes Wild) and the Natural Academy. Subsequent to this, a higher level six-day accredited Facilitator Training course was provided, with 16 clinicians attending. Training for using the NatureWell approach was provided by the Natural Academy and was based around the five pathways to nature connectedness: i) senses/ contact, ii) beauty, iii) emotion, iv) compassion, and v) meaning. The Natural Academy is a not-for-profit eco-social enterprise offering accredited training courses to individuals to enhance how they work with people to incorporate nature into their working style. The Nature and Health Facilitator training is designed for those working in health and social care and was delivered in 3 blocks with 4 days completed outside.

#### Data collection.

**Fig 1:**
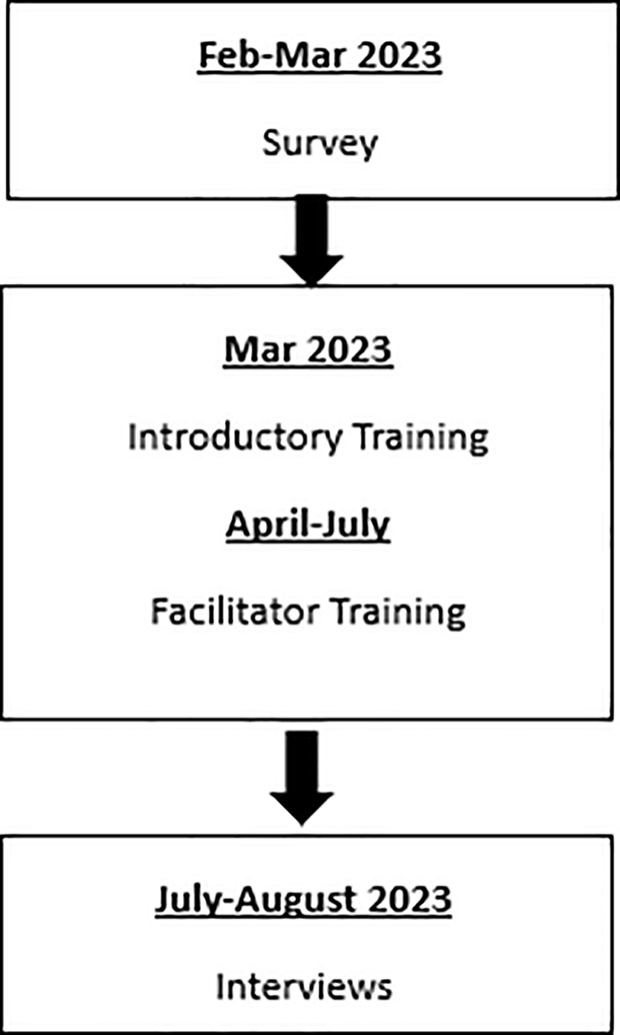
Study design flow chart.

Data were collected by the PenARC research team as part of a multimethod qualitative study which sought to understand staff experience and attitudes towards using NBAs in CAMHS. In this paper we report the findings from an online survey and 14 in-depth semi-structured interviews relating to the potential benefits of NBAs. The Natural Academy training described above took place between the survey and interview time points, see Fig 1. Survey findings were used to design the interview questions, with the aim to explain the mechanisms through which change was anticipated.

### Clinician survey

#### Sampling and recruitment.

All clinical and non-clinical staff working within the CAMHS service at one NHS Trust in the South of England were invited to participate in a short online survey administered through Qualtrics. Respondents were offered a £5 bank transfer, shopping voucher or a charity donation as compensation for their time. The survey covered respondent demographics and asked questions about staff experience and attitudes towards nature-based approaches. In addition, the survey assessed staff burnout, job satisfaction, and wellbeing, which are reported elsewhere [5]. The data reported here are from the free text responses collected on the anticipated benefits of using nature-based interventions in response to the question: ‘Can you think of any benefits to using nature-based interventions in your work?’.

#### Analysis.

Free text responses on anticipated benefits of using nature-based interventions were extracted from the survey data and analysed. Responses were read and grouped thematically by the lead author (BC) according to key words or concepts.

### Clinician interviews

#### Sampling and recruitment.

All CAMHS staff who completed the online surveys were invited to indicate if they would be happy to be contacted for an interview to explore the use of nature-based approaches in their work. Some (n = 8) had undertaken training with the Natural Academy as part of the CAMHS Goes Wild project and some (n = 6) had not. Of those who had completed the training, five of these had completed the Introductory Training only, and 3 had completed the Facilitator Training. Those interviewed were purposively sampled to ensure the data represented staff from a range of different professional roles within CAMHS (See Table 2), who worked in different settings with a range of patient groups and included both those who had attended the training and those who had not.

#### Interview procedure.

All participants provided written informed consent, including for audio-recording of the interviews. Semi-structured interviews took place between July and August 2023, by video call. All interviews were digitally voice-recorded and transcribed verbatim. Each recruited participant was assigned a unique identifier code. Interview recordings and transcriptions were stored on an encrypted hard drive. Once transcribed, interview data were managed using QSR International’s NVivo12 qualitative data analysis software (QSR International Pty Ltd., 2012) and were stored securely and password protected.

The topic guides for the interviews covered questions about clinician experience and attitudes towards integrating nature-based approaches (NBAs) in CAMHS. To elicit staff explanations about how NBAs might be beneficial in CAMHS, a realist informed approach was used in the development of the topic guides [38–40]. Interview questions were framed around understanding outcomes for staff and service users (e.g., What do you consider to be the outcomes or changes that using NBAs has had/may have for staff?), how outcomes may differ for different people (e.g., Do you think that the outcomes have been the same for all staff? In what ways have they been different?), potential mechanisms (e.g., We are very curious about how NBAs cause outcomes. How do you think the programme has caused, or helped to cause [one outcome identified by respondent]?), how mechanisms may differ by person or place (e.g., there are lots of ideas about how NBAs work, and we think they probably work differently in different places or for different people. One of those ideas is it gives staff more autonomy to be creative. Has it worked at all like that here or for you? Can you give an example?) and barriers or facilitators for implementation of NBAs (e.g., We’ve heard that NBA work differently in different places, what do you think it is about this place that makes it work so well/less well?). For full topic guide see S1 Text.

#### Approach.

Throughout the data analysis process researchers noted their thoughts on how to interpret the data and regular meetings of the research team provided space to reflect on and discuss these interpretations. This active construction of interpretations is important in reflexive research; whereby the researchers simultaneously construct interpretations (“what do I know?”) and questions how those interpretations came about (“How do I know what I know?”) [41]. The differing roles of the research team with regard to their involvement in the delivery of NBAs in CAMHS (BC is directly involved, all other authors work in a research-only capacity), meant that reflexivity was particularly important, acknowledging the experiences of the team to ensure that the themes generated were grounded in the evidence presented in the transcripts.

#### Analysis.

To ensure consistency and minimise researcher bias, strategies were employed throughout the analytic process. First, all interviews were read multiple times to facilitate familiarisation with the data, and transcripts were independently coded by multiple researchers, and discrepancies were discussed and resolved through consensus meetings, enhancing inter-coder reliability. Thematic development was iterative, with themes refined through ongoing discussion and reference to the raw data. The first stage of analysis, completed by BC and SMi, started with ‘indexing’ a small sample of interviews, to gather an insight and overview of the data. A thematic framework was then created, building on the initial review of data. This framework identified key concepts and was used to code all remaining interview data [42]. The next stage involved writing summaries of each interview for every code. This allowed for comparison, exploration and explanation of patterns [43]. This method facilitates systematic and transparent data analysis, and enables researchers to identify patterns or commonalities, as well as contradictions in and between participants’ accounts, so they can explore and test explanations for those patterns [44]. Refined themes were reviewed by BC, SMi and HB, and final themes and subthemes were then agreed [45].

#### Synthesis.

Synthesis of survey and interview data was completed using a process of triangulation [46] to explore potential benefits and challenges of NBAs in the context of CAMHS and potential mechanisms for these benefits. We do not explore potential challenges since these are described in a separate paper [37]. The method of triangulation describes the process of studying a problem using different methods to gain a more complete picture [46]. The process takes place at the interpretation stage of the study when both sets of data have been analysed separately. We have followed the triangulation protocol outlined in Farmer et al., 2006 which directs researchers to review codes and emerging findings from each component of a study alongside each other and to think about the meta-themes which cut across findings from different methods [47]. Data from the survey free text and the interviews were combined and the original themes were integrated, reviewed and refined to produce a final set of themes.

## Results

### Survey

Demographic data for survey respondents is presented in Table 1.

**Table 1:** Survey respondent demographics.

**Gender**	97 (83f, 13m, 1 gender fluid)
**Work pattern**	Full-time 59 (60.8%) Part-time 29 (29.9%) Missing 9 (9.3%)
**Role**	Other clinical: N = 36 (37.1%) Clinical associate psychologist: N = 16 (16.5%) Nurse: N = 12 (12.4%) Non-clinical: N = 12 (12.4%) Doctor: N = 8 (8.2%) Clinical Psychologist: N = 5 (5.2%) Psychology intern: N = 3 (3.1%) Other (occupational therapy, health and social care, etc.: N = 5). (5.2%)
**Age**	Under 25, N = 8 (8.2%) Between 25 and 34, N = 27 (27.8%) Between 35 and 44, N = 25 (25.8%) Between 45 and 54, N = 28 (28.9%) Between 55 and 64, N = 7 (7.2%) 65 and over, N = 2 (2.1%)
**Time at Trust**	Less than 1 year, N = 36 (37.1%) Between 1 and 2 years, N = 6 (6.2%) Between 2 and 5 years, N = 34 (35.1%) Between 5 and 10 years, N = 12 (12.4%) Between 10 and 15 years, N = 5 (5.2%) Over 15 years, N = 4 (4.1%)
**Attendance at training**	Both phases of training (introductory and facilitator training), N = 10 (10.3%) Phase 1 (introductory training) only, N = 38 (39.2%) No training, N = 49 (50.5%)

**Table 2:** Interview participant demographics.

**N**	14
**Role**	Clinical associate psychologist: N = 5 Education mental health practitioner: N = 4 Clinical psychologist: N = 1 Family therapist: N = 1 Nurse: N = 1 Assistant psychologist: N = 1 Not specified, N = 1
**Context**	Clinic-based: N = 9 School-based: N = 3 Mixed setting (i.e., school/home/clinic): N = 2
**Level of experience**	Management/Senior: N = 4 Mid-career: N = 4 Junior/Trainee: N = 5 Not specified: N = 1
**Attendance at training**	Both phases of training (introductory and facilitator training), N = 3 Phase 1 (introductory training) only, N = 5 No training, N = 6

#### Free text responses.

Of the 97 respondents, 77 submitted positive responses to the question “Can you think of any benefits to using nature-based interventions in your work?” Two responded with “no” and 18 did not submit any text. Four themes were identified (i) context enhancing benefits; (ii) enhancement of therapeutic intervention; (iii) Resourcing and connection; (iv) Health and wellbeing.

### Interview data

Fourteen interviews were conducted. Demographics of staff interviewed are detailed in Table 2. Interviews ranged from 30 to 52 minutes, with an average duration of 42 minutes.

**Fig 2:**
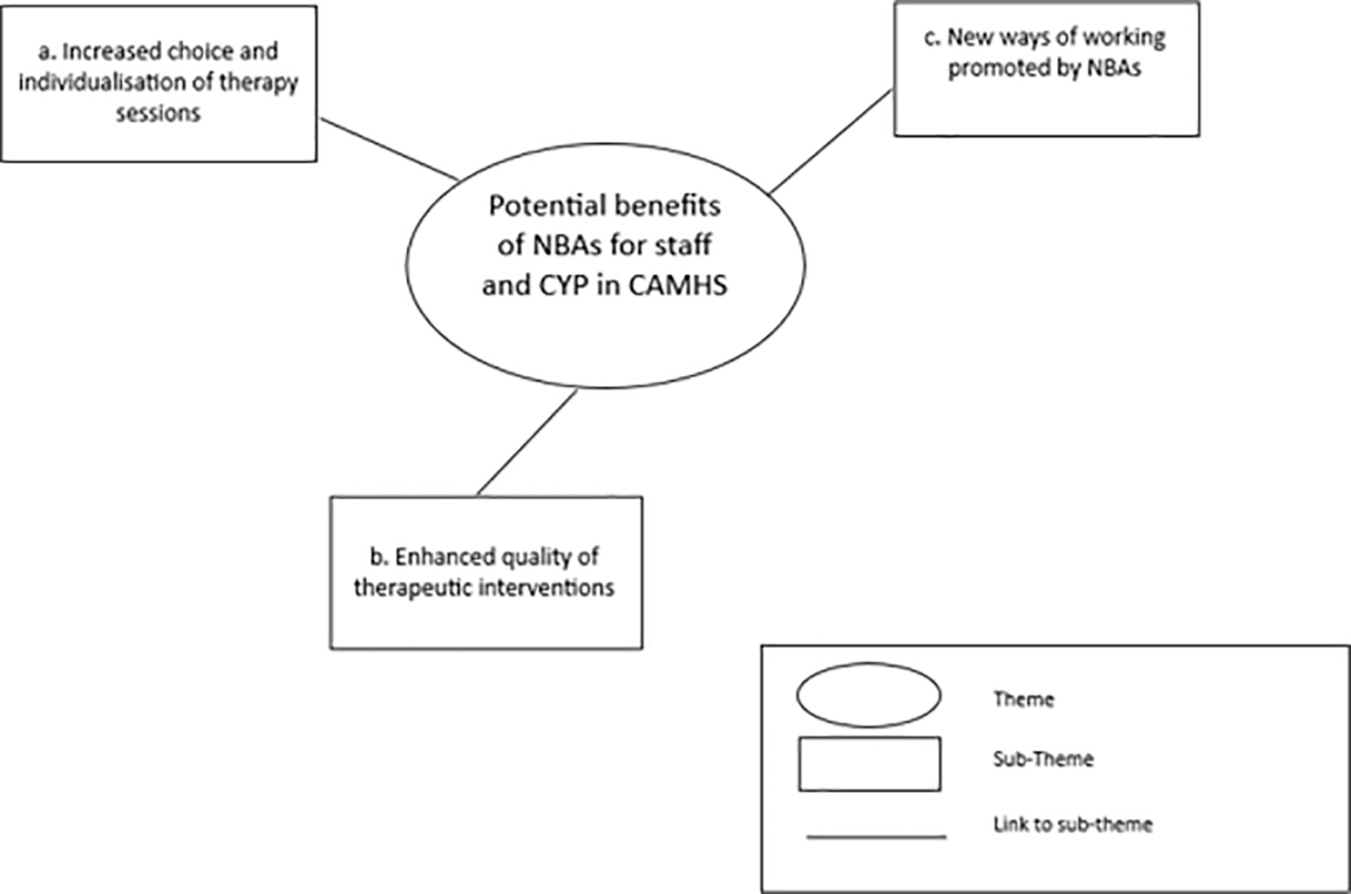
Thematic map.

Data synthesis generated three themes: Tension between the culture of CAMHS and NBAs (Theme 1) and the need for buy-in and governance support (Theme 2), whilst Theme 3 describes the potential benefits of NBAs for staff and service users in CAMHS and is the focus of this paper. The first two themes relating to the implementation of NBAs in CAMHS are presented in a previous paper [37]. Synthesis of survey and interview data to address the research question pertaining to whether staff consider that NBAs may be beneficial to service users and in what ways and circumstances, is illustrated by three sub-themes: Increased choice and individualisation of therapy sessions (a); Enhanced quality of therapeutic interventions (b); and new ways of working promoted by NBAs (c). See Fig 2. Staff reflected on the potential benefits of NBAs for both them and the CYP they work with. For some, this was a reflection based on training they had engaged with, for others this was a reflection on current or previous practice using NBAs in their work. Quotes are presented throughout from interview participants (P1-P14), and survey respondents (SP followed by participant number).

### Sub-theme a: Increased choice and individualisation of therapy sessions

Staff described how NBAs may benefit both staff and service users by offering more choice in how and where they have their therapy sessions. This may have important implications for engagement, both into services and into interventions.

Staff described the benefit of being able to offer greater choice to CYP which in turn may have implications for the engagement of CYP.

Mostly it’s more to do with having it as an option as a space to work in, so say if we’re working with a young person, making sure that we can meet them halfway, and if that’s something that they would usually be doing, is going out for a walk with mum and dad or going to the forest or something like that, that we could consider that as a place to meet them and we don’t have to be in a home or in a clinic, because sometimes those places aren’t very comfortable or aren’t very easy for our patients either. (P3)

Staff reflected that providing this choice facilitated learning opportunities, both for the CYP, in terms of decision-making, and for staff, in terms of supporting and facilitating decision-making.

I think everyone takes something different from it. I think some people really don’t want to go outside, they’re really like, “No, I’m not doing that.” Or they’ll do it now and again…But knowing that they have the choice and they have that control that they can go outside for a walk, they can get some fresh air. I’ll walk round with them, that’s fine, I don’t care if it’s raining, I’ll get wet. That ability to choose and to learn what feels okay in the moment, is important. (P1)

Having those conversations is a really good skill for staff to do anyway…proposing a plan is having a discussion. And not telling a young person how to then make a decision, like scaffolding them to learn how to make a decision, is a really good skill, so there’s lots of scope there…I guess part of it’s the clinician getting comfortable with it. (P2)

Staff described how offering choice can help CYP with their initial engagement into services: “…Well, these sessions can look how you want them to look. One of the things that some young people find beneficial is going outside” (P2).

It was felt that using NBAs could increase patient choice, person-centred care, accessibility to the service and promote inclusivity: “Young people being able to access support in a way which works for them-not every young person will feel able to access CBT and may find it challenging to talk about their emotions in a room” (SP67).

Hopefully that makes the sessions a little more informal, because I think a lot of young people might think, ‘I have to go to this therapy session, I really don’t want to talk about my feelings.’ But when you use a nature-based approach or a more informal approach, it doesn’t become that scary for young people…I think for them it offers support in a different way, not informally, may be more beneficial. I guess we’re able to use that person centred care because if people like beaches or people like a certain area, then maybe we can use that. (P10)

Staff also described how having NBAs as an option may help CYP to engage during therapeutic sessions and interventions and may also support the therapeutic alliance.

It’s the child’s choice if they want to be outside or not. They might be more comfortable inside, so I’d always have to double-check with them what they’d rather do…Maybe those children that are more – I don’t know what the right term is – less engaged maybe…and the less willing to share, a bit more closed off. It might be interesting to give them the choice to do a nature-based intervention session and help them relax into the sessions and bond to build up the therapeutic alliance. That could be really useful for those children. (P9)

Staff felt that this greater level of choice would be empowering not only for them but for CYP; potentially helping to improve attention and focus during therapy sessions, and enabling some CYP to stay engaged with the activities.

I think that really shows and we had one child that really, really struggled to focus. He loved the outside stuff. When it came to coming in and doing activities, his engagement was very limited and in fact, several times, the parent had to take them back outside, which we were observing…And, so he’d have that, calm down a bit, come back in, re-engage. Then, he’d maybe go to the, we had a “chillout zone” – he might go there for a bit and then, he had to go outside again. (P14)

I think it can help with their attention levels, their engagement if you’re outside in a park it’s easier for you to have a movement break so that’s a really different thing than actually using nature as the third thing. (P6)

In terms of the circumstances in which NBAs may be beneficial, staff felt that in some cases, NBAs could help to better meet individual needs, thus enhancing engagement and therapeutic benefits. Specifically, clinicians reflected that there may be benefits for the engagement of certain patient groups, including younger children, those with additional needs, neurodivergent CYP, those with low mood, and those who are socially anxious. For example, one interviewee said that the use of NBAs helped one CYP with mutism to express themselves via the use and variety of natural objects. Similarly, having sessions outside was described as more appropriate for some CYP with autism and ADHD, by reducing disruptive noises often found in clinical settings, such as echoey rooms and loud noises from adjoining rooms.

…working with kids that are autistic and ADHD, and have a lot of difficulties relating having those diagnoses. It’s going to be much more appropriate to get those kids outside so they can move and it’s not stressful, and the noises are just more natural noises rather than doors being slammed. The clinic rooms are not very sound proofed and autistic kids struggle in those rooms because they’re echoey as well. (P8)

It [NBA] took away the intensity because I think the mutism comes a lot from the intensity of people looking and talking in a school context, at home it’s a very intense environment. That freedom for him to express itself, not just verbally but through what he found, what he chose, the flowers he picked gave that variety that would be very difficult to provide resources for in a therapy room. (P11)

I think some of the young people we see often have low mood or depression, they don’t want to go out and they’re forced to come to these sessions, so I think if we can try and support them collaboratively, maybe just go outside, go to the park, go somewhere, hopefully that increases their mood as well so it won’t be another task for them to do when they get home. We also benefit if we could do that with them. (P10)

### Sub-theme b: Enhanced quality of therapeutic interventions

In addition to the potential to provide increased choice and to facilitate engagement, in both interview and survey data staff described how use of NBAs may be benefit CYP through enhancing the quality of the therapeutic intervention. Staff reflected how being outdoors makes CYP feel more relaxed (P9, P5, P2), and can improve low mood (P10, P13). This was also said to benefit staff members in similar ways (P9, P10).

I know that in my experience people have spoken mainly about just feeling more relaxed, and that the kind of peak anxiety of going to a CAMHS appointment, that peak has flattened a little bit when they know that it’s more of a walk and talk, or we’re going to do something that’s a lot more indirect than being fired questions at, which can feel like an interview in a dentist-looking room. All of those things are counterproductive for therapy. (P2)

I see that in children as well when I’ve worked in schools, being outside and having that freedom being around nature is quite good and it relaxes children as well…Something as simple as just sitting outside on the field or on the bench doing a session…I did notice some differences when we did it outside…Noticed it myself that I probably enjoyed the session, I felt more relaxed, and also the child made quite a lot of progress in that session because I think they were more relaxed as well…” (P9)

Being in a more relaxed state can enhance engagement and effectiveness of the therapy session: “young people that I’ve worked with in the clinic space, when we’ve gone outside they’re more verbal.” (P11) and “Many find the clinic room environment to be repressive and deflating, which can impact upon the effectiveness of consultations” (SP8).

Staff described the context enhancing benefits of NBAs. Staff contrasted being outside with sitting in a traditional clinic environment (sometimes without windows) as positively impacting on the mental state of clinicians and patients and potentially improving outcomes: “My clinic room has no windows and rarely drops below 26 degrees. The walls are white (we are not allowed to hang anything). You can imagine how a day in there makes you feel!” (SP10).

Staff identified it bringing a sense of calm and safety through reducing perceived threat to promote healing and an overall positive CAMHS experience: For example, “Improved calmness and clarity for patient and clinician - so more difficult subjects can be tackled” (SP50).

When you go for a CAMHS appointment there’s all kinds of bad feelings going on, so if you can have that appointment in a space that has got some level of diversity, fresh air, all that kind of stuff, it’s helpful for some of our clients. Some clients find that space [clinic] challenging as well. (P2)

NBAs were considered to facilitate talking (especially around difficult topic areas) and tolerating silence. The nature-based environment was referred to as a “second therapist” providing new perspectives and a freedom of space and movement which can reduce the intensity of therapy.

[NBAs provide] …a more calming safe space for them to share, because we’re talking about quite personal things, so if you remove them from the school, not the premises but from that place. Because a lot of the children that I work with have worries and anxiety linked to school, so what happens in the school, so if you remove them just slightly from that it might help break down some barriers. (P9)

NBAs were described as being able to promote a more informal environment with positive conditions for person-centred work, interaction, creativity, movement, and relaxation. In turn, practitioners reflected that this environment could enhance the therapeutic process. For example, supporting regulation, and facilitating CYP opening up and helping with the flow of conversations.

Clinic areas can feel quite formal, so when we’re coming to things like disclosures and talking about trauma. I’ve definitely worked with young people who have felt like I could say it on a walk, if I say it in this room it feels like it’s heavier and it means more and it’s more serious and there’s consequences for it, and that makes it way scarier…There’s a lot of lightening that can be had just in the change of environment that can really open up a lot of doors that I think get closed off as soon as you literally walk into a clinic room. Part of the emotional burden that we hold as therapists is to try and dismantle all of those barriers that get put up in the environment around us, so I imagine there’s some liberation there both for the therapist and for the young person and being a little bit more open and just kind of going with being outside…I think it’s transferable skills and integration of psychological skill into external places. (P2)

Staff described how using NBAs could enhance specific aspects of therapeutic work such as emotional regulation and grounding; mindfulness; behavioural activation; and sensory integration: “Supporting behavioural activation work, mindfulness, good for both practitioner and client” (SP30) and “Access to nature supports emotional and sensory regulation” (SP65).

Staff reflected that using a parallel activity such as walking or choosing natural objects can take the pressure off the conversation:

…just general walking out, often walking is a nice parallel activity especially with young people that struggle with sitting down and staring at the questions sometimes having walking can take the pressure off the conversation and make them feel a bit more open. (P13)

…I think if you’re working more with a lot of those client groups [CYP with ADHD, Autism], as one of my colleagues I’m thinking of is, then she does go outside loads and I think it really helps when you’re walking and talking, rather than sat in a clinic room staring at somebody asking them questions. (P8)

They also felt it could positively impact on the therapeutic approach by reducing pace, appearing less hierarchical and more informal. They felt this could make clinical interactions more “informal and relaxed, less intense, fun” (SP90) for CYP and clinicians in addition to increasing motivation, participation, engagement and rapport.

### Sub-theme c: New ways of working promoted by NBAs

Staff described how the use of NBAs promoted new ways of working which facilitated increased autonomy, creativity and flexibility both to the benefit of staff and service users. These benefits were described as longer term and beyond the scope of individual therapy sessions.

A more flexible and creative practice afforded by using NBAs was described as having the potential to benefit staff skill, enjoyment, job satisfaction and confidence: “…feels more useful than being in the clinic room, so I suppose that might help you feel better at your job” (P8) and “I think it gives permission to be creative, job satisfaction, that enjoyment, that being able to be flexible to where you are, where you’re delivering and also, the child’s needs” (P14).

Staff described a multitude of benefits they felt that working in this way could facilitate including learning new skills, having more fun, variability at work, and working in a way that aligns with their own values.

It would help to retain me in my role, it would be a chance to learn some new skills, the environment would make me happier at work it would have benefits for my own physical and mental fitness, it would be more fun and enjoyable with the age group I work with (SP18)

Staff reported that when working in this new way they would feel more empowered to work more autonomously, and that working creatively increases enjoyment.

I think it’s empowering. I think it could increase the confidence of practitioners who maybe feel quite intimidated by the concept and managing the risk of taking a young person into nature. I also think it’s a really healthy way of supporting staff to diversify skills in the way you approach your clinical appointment. Like anybody else, if you do something over and over again you can fall into a pattern and a dynamic of doing it, and going outside in nature challenges us to go against that a little bit. I think in that space you can be more creative and you can become more confident and more flexible in your approach, which I think is really healthy, and challenges clinical drift which can happen if you get stuck in those patterns. (P2)

Definitely in my role when I go into schools, I don’t have that much control where we sit, where we are, what we have around us, so I like that control of being outside definitely with that autonomy and that freedom as well, yeah. (P9)

Staff reflected that the additional flexibility and creativity afforded to them through working in this way may in turn benefit CYP in terms of their own autonomy. Employing NBAs was described as a way to provide CYP with additional coping skills, and opportunities to apply these skills in external spaces (as opposed to the clinic room), thereby enhancing the therapeutic work and increasing autonomy: “Offering theories into practice, for instance mindfulness practice while on the beach” (SP53).

Staff described how NBAs could have a positive effect on the general health and wellbeing of clinicians and patients. Suggested pathways included stress reduction and a sense of fun; improving mood, self-esteem and self-confidence; increasing hopefulness and a sense of purpose: “I would be a much happier practitioner if I could deliver outdoor interventions as my role. I find being stuck in bland stuffy clinic rooms stressful and uninspiring” (SP15) and “Improving my own wellbeing as well as those I am working with due to being immersed in nature” (SP28). Bio-physiological improvements in blood pressure, heart rate, immune system, serotonin, and vitamin D levels were also noted: “Sunshine provides vitamin D which strengthens the immune system and decreases stress, also reduces blood pressure. Being outdoors encourages physical activity” (SP27).

Being out in nature may provide CYP with an opportunity to apply and utilise skills such as grounding, equipping them with transferable skills and an opportunity to apply them, thereby enhancing the utility of therapeutic interventions.

If they are anxious being out socially but they enjoy going to the beach, you can do it with them and then give them a bit more autonomy and I guess confidence for them to be able to do it themselves as a coping tool. (P10)

This experience may enhance feelings of autonomy and wellbeing outside the therapeutic space, empowering CYP to apply versatile techniques and develop coping skills that are more easily integrated into their daily lives: “I think it’s transferable skills and integration of psychological skill into external places” (P2).

I think that was really important as part of her realising that she doesn’t need to be dependent on therapy and treatment, and that she’ll be okay, she’s got ways to cope and to manage. If she’s feeling stressed at home she can go for a walk… I think those are skills that are so important, because we need to be helping young people transition from relying on a therapist to help them regulate, to learning how to regulate themselves within their environment (P1).

Learning to take what you learn inside a room with one person that you meet infrequently and apply it to the rest of your life, is a really big skill. To generalise that skill, to take ownership of these therapeutic skills that you learn in a treatment and actually even think about applying them to your life, let alone actually implementing them, is a big ask, and we ask that of young people so that’s really hard…I think taking a therapy session outside is kind of a symbolic way of demonstrating that and showing that you could do this in this part of your life, in this slice of your life, and it breaks down what I think is quite a big barrier between what you do in therapy and the rest of your life, and we need to integrate that anyway. We try and integrate it by talking to them and saying, “You need to do this at home,” but that sounds like homework and no-one really wants that. Whereas literally moving outside is an active way of modelling what we’re talking about when we’re talking about integrating that work into the outside (P2).

Staff felt that NBAs could increase connectedness to nature and the environment; to a sense of place and other; to family; and to one’s own body. This could resource the young person with new skills and coping strategies that can be continued after recovery and accessed independently of CAMHS: “The children and young people will benefit and so will families as a whole- it promotes connection with each other as well as nature, opportunities to learn and develop skills including regulation and containment” (SP89).

They also noted how using NBAs could provide an opportunity to model behaviours to families, such as promoting creativity, the use of local natural resources, good self-care and positive risk taking.

Being in nature can be calming to some young people and is accessible, this can help promote sustained improvements to mental health especially living in a predominantly rural community like Cornwall where there is access to green and blue spaces (SP16)

## Discussion

The findings from this study elucidate some of the potential benefits of adopting NBAs in CAMHS, the ways in which NBAs might enable those benefits, and the circumstances in which NBAs may be particularly beneficial, from the perspectives of CAMHS staff. Potential benefits described by participants extended beyond service users to staff members, suggesting the possibility of more wide-reaching benefit from individual to service level. Potential benefits centred around NBAs affording greater choice and individualisation; enhancing the quality of therapeutic interventions; and promoting new ways of working which were perceived to offer greater autonomy, creativity and flexibility for staff and CYP. All of these were considered to positively impact on health and wellbeing.

Reviews of the benefits of nature connectedness for CYP have identified specific patient groups who may benefit, such as those with behavioural difficulties including hyperactivity and inattention problems [23,24]. Our study builds on this finding by explaining why and how nature connectedness may work for these CYP. Staff highlighted that nature-based approaches may be particularly beneficial for young people with additional needs, neurodivergence, low mood, and social anxiety. They explained that NBAs may provide opportunities for a more individualized and flexible therapeutic experience, allowing young people to engage in a way that feels more accessible and comfortable. For example, staff noted that nature-based options can foster engagement by offering a less intimidating environment compared to traditional clinic settings or face-to-face conversations. Consistent with Hunt et al. (2022), this approach may also strengthen the therapeutic alliance, with the element of choice serving as a crucial foundation. A unique insight from this study was the recognition of the decision-making process as a key factor in both CYP and staff development. Offering young people choices in therapy was seen not only as empowering for CYP but also as a valuable skill-building opportunity for staff in supporting these choices effectively.

While relatively little research has been conducted in this area, Hunt et al. (2022) identified the potential of NBAs to support therapeutic outcomes like emotional regulation. Building on this, our study suggests that NBAs may have the potential to enhance the quality of therapeutic interventions in a number of ways, including emotional regulation, grounding, mindfulness, behavioural activation, and sensory integration. Being in nature activates the parasympathetic nervous system which is associated with a sense of safety and stress reduction [10]. This can create a sense of grounding which can make therapy more accessible rather than entering into it from a heightened state. Parallel activities such as walking and connection with nature were described as components which could help to enhance the therapeutic process because they can shift the focus away from the young person, facilitating conversation and observation of transferable metaphors in nature which may feel less ‘threatening’ than talking about one’s own vulnerabilities. Equally, the sensory aspects of being in nature were described as helpful to facilitate mindfulness activities, offering rich sights, sounds and smells. In both examples provided, nature is acting as a ‘second therapist’. In order to realise these benefits, existing research on the implementation of NBAs in the CAMHS setting highlights the importance of clarity for staff around the form and function of NBAs in this context [37]. Future studies and practice should prioritise explication of what these new ways of working look like in practice to promote the adoption and implementation of NBAs in CAMHS.

These views fit with the proposed theoretical mechanisms for the benefits of being in nature. The experience of Attention Restoration could be advantageous for those who struggle more to stay focused within a clinical room such as those with inattention. In this situation integrating elements of nature into therapy or changing the environment of therapy, from clinical room to outdoors could enable this mechanism, restoring attention and engagement in the therapeutic process. The reduction in physiological and subjective experience of stress when in nature was noted by respondents to be potentially universally helpful and aligns with how the parasympathetic nervous system is known to respond when in nature [48]. This may underly staff views that CYP may be more able to engage with therapy and that approaches such as emotional regulation skills may be more effective when using NBAs as arousal levels are already reduced.

Staff highlighted that NBAs could resource CYP with valuable skills and coping strategies that extend beyond their recovery and can be utilised independently of CAMHS. They noted that NBAs offer a chance to model positive behaviours for families, such as fostering creativity, utilizing local natural resources, practicing good self-care, and engaging in positive risk-taking. This supports Hunt et al. (2022), who described NBAs as fostering a sustainable approach that encourages the use of skills beyond the clinic setting, aligning with recovery-oriented practices commonly used in inpatient settings [49]. From a recovery standpoint, this is significant, as it emphasises the importance of therapeutic practices that help service users apply coping skills in real-world contexts, enhancing their practical utility outside of the clinical environment. This also aligns with the Biophilia Hypothesis, that we are hardwired to form relationships with and connections to nature and perhaps what is missing for our CYP is opportunity to do this [9,10]. By modelling this approach using NBAs we are reconnecting our CYP and their families with nature.

The new ways of working promoted by NBAs that involve working outside the traditional clinical setting and offering greater flexibility, autonomy, and creative freedom, were described as beneficial for staff as well as service users. Previous studies suggest that NBAs can help reduce staff burnout, with research from Australia indicating that clinicians found NBAs to bring personal benefits for themselves, experiencing NBAs as therapeutic, relaxing, and refreshing [36]. Similarly, staff in this study, while prioritising the benefits to service users, also noted that the flexibility and creativity inherent in NBAs could enhance their own wellbeing. The increased autonomy associated with NBAs was highlighted as advantageous for both staff and service users, aligning with broader literature that links autonomy to improved wellbeing [50,51]. Alongside the physiological benefits commonly reported in research, staff also mentioned potential gains in job satisfaction, skills, enjoyment, and confidence. In a sector where staff wellbeing is reported as poor [e.g., 5], and staff retention as low [52], this is an important finding which warrants further exploration.

While this study has highlighted the potential benefits of this approach, there are also many practical challenges which may impede the adoption and implementation of NBAs in the CAMHS setting [37]. Specifically, when it comes to NBAs, existing research highlights the potential for conflict between the prevailing risk-averse culture of CAMHS and NBAs, which inherently promote positive risk-taking [37]. At a cultural level, risk perception in mental health services is oriented towards risk reduction, while this approach necessitates viewing risk differently [53]. That said, research suggests that at an individual level, many staff are open to new ways of working [37]. Further, staff described a need for consideration of, but not focus on, risk and the need for organisational support to enable the flexibility required to share or dynamically assess risk [37]. Other facilitators described in relation to enabling implementation of NBAs in the CAMHS setting included buy-in and governance support, such as harnessing the role of firsthand experience, involvement of a range of stakeholders in the adoption and implementation process, and demonstration of support from management [37].

### Strengths and limitations

The multimethod approach provided a comprehensive and robust way to address the research question, enabling us to gather and triangulate a wider range of perspectives from staff across the service. Participants had a variety of different backgrounds and experience of using NBAs. It is possible that the self-selected sample were influenced by their own positive experiences of being in nature. Triangulation enhances the validity of our findings through the convergence of evidence from multiple sources and perspectives. This supports mitigation of bias and the limitations inherent in any single approach, leading to a more robust and trustworthy understanding of the research topic. This study utilised qualitative, self-reported data. To understand a wider variety of perspectives on this topic and to broaden the applicability and enhance the validity of findings, future studies should consider alternative methodologies and designs. This research also came at a time when the service was just beginning to consider how NBAs could be implemented in care provision and the palpable enthusiasm around this possibly enhanced participants’ positivity around NBAs. The time between training and the interviews was too short for staff to be able to reflect on any meaningful change that may have occurred in service delivery and using NBAs. Therefore, it is likely that interviews only picked up data that were linked to pre-existing ways of working or their hypothetical thoughts about how NBA could be beneficial. This research also only focussed on staff attitudes and did not consult with users of the CAMHS service. Engagement with children, young people and families is a vital next step to ensure that this approach is one that would be acceptable for them as well as the clinicians delivering care. As far as we are aware this is the largest study exploring staff views of working in a nature-based way, however, it only provides a snapshot of staff attitudes and further research, conducted over an extended timeframe, which includes service user perspectives would be needed to determine if perceived benefits do indeed translate into improved outcomes for service users and staff.

## Conclusion

This study highlights the potential benefits of adopting NBAs in Child and Adolescent Mental Health Services (CAMHS), extending beyond service users to also positively impact staff. The findings suggest that NBAs can enhance therapeutic interventions by offering greater choice, individualisation, and flexibility, which may improve engagement and inclusivity for young people, particularly those with specific needs such as neurodivergence, low mood, or social anxiety. The study also points to the benefits of NBAs in fostering a stronger therapeutic alliance and providing a diverse set of therapeutic tools, such as emotional regulation and mindfulness, with nature itself acting as a supportive element in therapy.

Moreover, the adoption of NBAs may offer substantial benefits for staff, including increased autonomy, creativity, and job satisfaction, which are crucial in a sector where staff wellbeing and retention are ongoing challenges. This way of working may not only contribute to better therapeutic outcomes but could also support staff wellbeing by reducing burnout and enhancing the overall work environment. In a high stress, low predictability work environment, the unique benefits being offered by the natural environment may be of particular benefit for staff. Additionally, NBAs offer a sustainable approach to patient recovery, equipping young people with skills and coping strategies that can be applied outside the clinical setting, thereby increasing the potential for long-term resilience and real-world utility. These findings underscore the importance of further research into NBAs to explore their full potential in enhancing both service user and staff experiences in CAMHS.

## Supporting information

S1 Text Interview topic guide. (DOCX)
